# Recent Applications of Near-Infrared Spectroscopy in Food Quality Analysis

**DOI:** 10.3390/foods13162633

**Published:** 2024-08-22

**Authors:** Mohammad Nadimi, Jitendra Paliwal

**Affiliations:** 1 Department of Biosystems Engineering, University of Manitoba, Winnipeg, MB R3T 5V6, Canada; mohammad.nadimi@umanitoba.ca; 2 Department of Applied Computer Science, University of Winnipeg, Winnipeg, MB R3B 2E9, Canada

With the ever-increasing global population, food demand will continue to increase in the coming decades. This unprecedented demand for food necessitates effective quality monitoring to minimize loss and ensure a safe and secure food supply. Since production cannot be increased indefinitely, the food industry must adopt reliable technologies to minimize post-production losses. Over the last two decades, sensor technology advancements combined with chemometrics have established near-infrared spectroscopy (NIRS) as a promising technique that offers a non-destructive and rapid solution for food quality monitoring. However, the translation of lab-scale research to commercial solutions still evades the agri-food sector, and further research is required to realize NIRS’ full potential.

This Special Issue of *Foods* covers a broad spectrum of applications where NIRS has shown unparalleled potential in a variety of agri-food products as they move from producers to consumers post harvest. These include quality changes in cereals, oilseeds, legumes, tubers, and fish due to sub-optimal storage and handling. This Special Issue also illustrates how NIRS is enabling the exploration of new avenues in determining the quality parameters of manufactured foods such as baked, processed, and extruded products. Further advancements of NIRS, such as providing the spatially resolved information of interrogated samples, commonly known as hyperspectral imaging (HSI), have also been highlighted. Researchers are developing novel ways to sift through large datasets using chemometrics, artificial intelligence, and non-linear data mining techniques. This eclectic curation of cutting-edge research studies provides researchers with a platform for sharing groundbreaking novel findings and insights on the recent applications of NIRS in various food domains.

In this context, Indore et al. [1] utilized HSI (in the visible to near-infrared (Vis-NIR) and the short-wave infrared (SWIR) range) and synchrotron phase-contrast micro-computed tomography (SR-µCT) to assess quality changes in Kabuli chickpeas during storage. The authors analyzed chickpeas with various moisture contents (ranging from 9 to 15% wet basis) at three different temperatures (ranging from 10 °C to 30 °C). Utilizing multivariate statistical techniques such as principal component analysis (PCA) and partial least squares discriminant analysis (PLS-DA), the study successfully classified samples based on relative humidity and storage temperatures, with a classification accuracy of 80–99%. Moreover, SR-µCT analysis revealed microstructural changes at higher temperatures (above 20 °C). These findings suggest that HSI can potentially be used for legume quality monitoring during storage.

In another work, Olakanmi et al. [2] utilized HSI to predict the protein content and classify the color characteristics of fava bean-fortified bread. The study employed SWIR and Vis-NIR ranges to assess protein distribution and color quality. Using multivariate analysis tools such as PCA, PLS-DA, and partial least squares regression (PLSR), the authors achieved a classification accuracy of over 99% for protein content. The work indicates that HSI can effectively classify bread samples based on protein content and predict protein composition, making it a reliable tool for quality monitoring in commercial bakeries.

Nadimi et al. [3] explored the potential of HSI combined with chemometrics to characterize mechanical damage and assess germinability in flaxseeds. The study evaluated flaxseed samples at varying moisture contents (6–11.5%) subjected to different impact stress levels (0–6 mJ). Hyperspectral images were acquired across Vis-NIR and SWIR ranges. Using PCA and PLS-DA, the authors achieved more than 90% overall accuracy for the incurred mechanical damage. PLSR models predicted germinability with R^2^ values of 0.78–0.82. These findings suggest that HSI is a promising approach for assessing mechanical damage and germinability in flaxseeds.

In another work, Peiris et al. [4] utilized a handheld MicroNIR (VIAVI MicroNIR OnSite-W (VIAVI Solutions Inc., Santa Rosa, CA, USA)) instrument to identify sorghum grain protein levels. Near-infrared calibrations were created using an established benchtop and adaptive handheld instruments. Spectra from samples were collected by both instruments. While the benchtop instrument demonstrated superior performance with an R² of 94% on the test set, the handheld MicroNIR also showed acceptable accuracy, with an R² of 87%. The handheld device’s performance suggests it is a viable, less expensive tool for field applications where the utilization of benchtop instruments might be challenging.

Fodor et al. [5] utilized the Fourier transform near-infrared (FT-NIR) (BRUKER, Ettlingen, Germany) technique combined with chemometric methods to determine the ripeness of plum samples non-destructively. The study focused on key quality parameters, including dry matter (DM), titratable acidity (TA), total soluble solids (TSS), and the calculated maturity index (MI: TSS/TA) as reference values. Various machine learning algorithms, including linear discriminant analysis (LDA), quadratic discriminant analysis (QDA), and Mahalanobis discriminant analysis (MDA), were applied to predict the plums’ TA, TSS and MI, achieving 100% accuracy in classifying plums at different maturity levels. This work suggests that FT-NIR, combined with robust chemometric methods, is a highly accurate and efficient tool for assessing plum maturity.

Cho et al. [6] applied SWIR HSI to classify the freshness of mackerels. To develop a predictive model, they analyzed total volatile basic nitrogen (TVB-N) and acid values, which are chemical indicators of freshness. Fresh mackerels were categorized into three groups based on storage periods (0–48 h), and hyperspectral data were collected from the eyes and whole body separately. The prediction accuracy for TVB-N was 91%, and the acid value was 84%. These findings demonstrate that HSI can potentially verify the freshness of mackerels and predict associated chemical compounds.

Khorramifar et al. [7] investigated the changes in pH and soluble solids content (SSC) of potatoes during storage using Vis-NIR spectroscopic techniques (Model PS-100; Apogee Instruments, INC., Logan, UT, USA) and an electronic nose (E-nose) (the most common commercial sensor). Various prediction models, including partial least squares (PLS), multiple linear regression (MLR), principal component regression (PCR), support vector regression (SVR), and an artificial neural network (ANN), were used to assess these changes. The PLS model exhibited the best performance in Vis-NIR spectroscopy for predicting SSC and pH, achieving respective accuracies of 89% and 93%. The implementation of the ANN in analyzing E-nose data led to 83% and 94% accuracy in predicting SSC and pH, respectively. These findings suggest that Vis-NIR spectroscopy can potentially be used to monitor potato quality during storage in a non-destructive manner.

While the advancements in NIRS and associated chemometric techniques presented in this Special Issue are promising, several limitations must be addressed to realize their full potential. One major limitation is the need for extensive calibration models specific to the type of food and its storage conditions, which can be time consuming and require large datasets. Additionally, the accuracy of NIRS can differ when utilized in real-world commercial facilities rather than in controlled lab settings. Utilizing artificial intelligence tools can help address some of these challenges. Moreover, developing models with samples that exhibit greater variance can enhance the robustness and applicability of NIRS technology. Future research should also focus on developing more robust and generalized calibration models that can be easily adapted to different types of food and varying conditions. Moreover, developing portable and user-friendly NIRS devices will be crucial for field applications, making this technology accessible to a broader range of users, including small-scale farmers and food processors.

Overall, the studies featured in this Special Issue demonstrate the remarkable potential of NIRS and chemometric methods in food quality and safety analysis. Continued research and technological advancements will further enhance the applicability and effectiveness of these methods, contributing to a safer and more sustainable food supply chain.
